# Model Analysis of Fomite Mediated Influenza Transmission

**DOI:** 10.1371/journal.pone.0051984

**Published:** 2012-12-27

**Authors:** Jijun Zhao, Joseph E. Eisenberg, Ian H. Spicknall, Sheng Li, James S. Koopman

**Affiliations:** 1 Institute of Complexity Science, Qingdao University, Qingdao, China; 2 Department of Epidemiology, University of Michigan, Ann Arbor, Michigan, United States of America; 3 Center for the Study of Complex Systems, University of Michigan, Ann Arbor, Michigan, United States of America; Yale School of Public Health, United States of America

## Abstract

Fomites involved in influenza transmission are either hand- or droplet-contaminated. We evaluated the interactions of fomite characteristics and human behaviors affecting these routes using an Environmental Infection Transmission System (EITS) model by comparing the basic reproduction numbers (*R*_0_) for different fomite mediated transmission pathways. Fomites classified as large versus small surface sizes (reflecting high versus low droplet contamination levels) and high versus low touching frequency have important differences. For example, 1) the highly touched large surface fomite (public tables) has the highest transmission potential and generally strongest control measure effects; 2) transmission from droplet-contaminated routes exceed those from hand-contaminated routes except for highly touched small surface fomites such as door knob handles; and 3) covering a cough using the upper arm or using tissues effectively removes virus from the system and thus decreases total fomite transmission. Because covering a cough by hands diverts pathogens from the droplet-fomite route to the hand-fomite route, this has the potential to increase total fomite transmission for highly touched small surface fomites. An improved understanding and more refined data related to fomite mediated transmission routes will help inform intervention strategies for influenza and other pathogens that are mediated through the environment.

## Introduction

Influenza transmission occurs through various environmental routes [1]–[5], such as aerosol (where infection occurs through inhalation of droplet nuclei, the shrunk dried droplets with diameters<10 µm) [6]–[10], droplet spray (where particles with diameters larger than 10 µm from a cough or sneeze directly deposit on the mucous membranes of others), direct contact (where pathogen transfer occurs via a handshake) and indirect contact (where pathogens transfer from fomites to hands) [11], [12]. Previous studies have highlighted the importance of the ‘indirect contact’ route [12], [13]. Uncertainty analysis found that despite the relatively fast inactivation of influenza virus on hands and surfaces, contact transmission remains a viable transmission route, in part due to the vast volumetric majority (99.99%) of cough excretions being so large that they settle from the air rapidly [4], [14]. Due to this result, as well as the increasing interest in hand hygiene and decontamination to control a wide range of infectious diseases we focus here on the indirect contact route. Specifically, we examine the effects of human behavior on fomite contamination and in turn how fomite characteristics affect the fomite mediated transmission.

Fomite contamination can occur either: 1) through droplet deposition from coughing, sneezing, or exhaling; or 2) through deposition from contaminated hands. To understand how the source of contamination affects transmission, we extended our Environmental Infection Transmission System (EITS) model [15] to include two different fomite mediated transmission routes in one venue. We define the two fomite mediated transmission routes as the:

1. Droplet-contaminated-fomite route: exposed surfaces near an infected person become contaminated by spray or settling of virus laden large droplets.
2. Hand-contaminated-fomite route: hands of infected individuals are contaminated with virus following excretion; virus is then deposited to surfaces.

For both routes the path of transmission subsequently involves susceptible individuals contaminating their hands and self-inoculating virus to their mucosal surfaces. By separating fomite routes based on the contamination source, we can study effects of human behaviors (e.g. covering coughs by hands) and fomite characteristics on fomite mediated transmissions.

Previous studies have characterized fomites based on either: 1) materials with different transfer efficiencies and die-off rates (e.g., porous or nonporous) [6], [16], or 2) frequency of being touched [15]. Neither of these studies assessed the relative transmission contributions of different types of fomites in a venue. In this manuscript we classify fomites by parameter values of surface sizes and touching rates; we model fomite types separately as if each has an independent contribution to the basic reproduction number, *R*_0_. This tactic allows us to mathematically separate how different fomite routes and fomite types influence influenza transmission, while elaborating on what affects the relative contributions of each fomite type and route.

By mathematically analyzing the model with only two fomite routes, we demonstrate first, that fomite mediated transmission is more likely to involve droplet contamination than hand contamination; and second, that the highly touched large surface fomite type has the highest transmission potential. We conclude by identifying parameters that have strong effects on transmission, thereby elucidating potential valuable intervention targets.

## Methods

We model the fomite mediated transmission in a venue that has two fomite transmission routes and on four types of fomites. We make the following assumptions about the environment:

- Fomites are defined as those surfaces and materials in the environment that can be touched by human hands; we exclude surfaces that under normal circumstances are never touched by human hands such as floors, ceilings etc.
- Populations touch portions of the fomite homogeneously, and pathogens on fomites are homogeneously distributed.
- The total population size *N* is constant (*N* = *S + I + R*), where *S*, *I, R* are the numbers of susceptible, infected and recovered individuals.
- Transportation of contamination from one type of fomite to another via human hands is not modeled, i.e., each class of fomite is independent.
- Pathogens on hands are located on fingertips.

All model assumptions in [15] apply to our extended EITS model.

**Figure 1:**
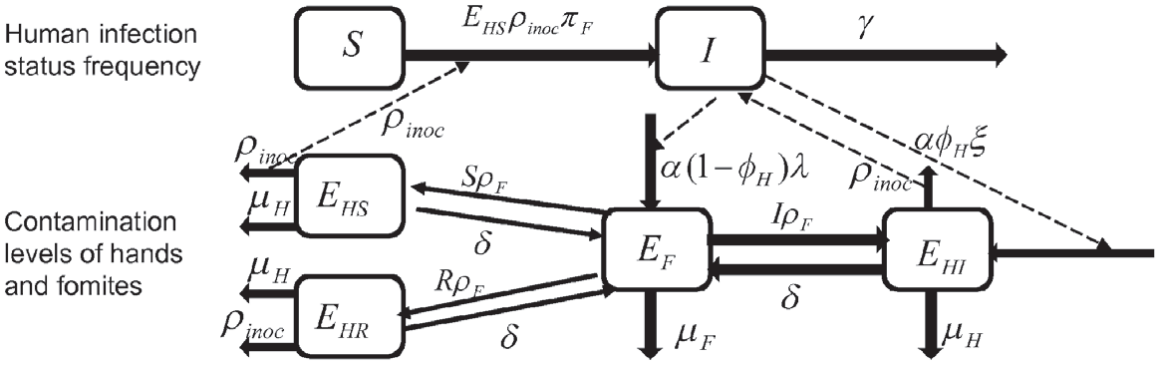
Compartments and transmission routes. The upper part of the figure models the flow of individual compartments. The transfer rate from susceptible (*S*) to infected (*I*) is the product of the pathogen amount on hands of susceptible (*E_HS_*), the fraction of pathogens *E_HS_* transferred to mucus of susceptible via inoculation (*ρ_inoc_*) and the probability that a susceptible individual becomes infectious per pathogen. The recover rate of infected is *γ* per unit time. The lower part of the figure models the flow of pathogens. *α* is the shedding rate per unit time per individual. *φ_H_* proportion of virus goes directly to the hands of an infected individual. The proportion of virus that is excreted to hands and then is associated with a type of fomite is *ξ*. *λ* is the fraction of pathogens going to surfaces reach one type of fomite. *ρ_F_* is the individual pick up rate of pathogens on a fomite per unit time and *δ* is the deposit rate of pathogens from hands to fomites per unit time. *µ* *_H_* and *µ* *_F_* are virus inactivation rate on hands and fomites respectively.

First we describe the model of the transmission process defined by one fomite type, and two fomite contamination routes (droplet versus hand). This ordinary differential equation based model has 6 compartments: susceptible humans (*S*), infected and infectious humans (*I*), pathogens concentration on hands of susceptible (*E_HS_*), infected (*E_HI_*), and recovered (*E_HR_*) individuals, and pathogens on fomites (*E_F_*). Figure 1 summarizes the compartments and the two transmission routes. Model parameters are summarized in Tables 1 and 2.

**Table 1:** Parameter Values Used in Comparison of *R*_0_s.

Parameters	Parameter Description	Value	Range	Unit	Reference
*N*	Population size	1,000	[100, 6,000]		
*ρ_inoc_*	Inoculation rate	0.08	[0.02, 0.3]	1/person/minute	[4], [18], [19]
*α/γ* a	Shedding amount	1×10^7^	[5×10^6^, 1.5×10^7^]	Pathogens/infected	
*π_F_*	Dose response of virus on mucosa	6.93×10^−5^	[0.001, 1.4×10^−4^]	Infections/pathogen	[15]
*µ_H_*	Virus inactivation rate on hands	1.2	[0.01, 3]	1/minute	[17]
*µ_F_*	Virus inactivation rate on surfaces	0.01	[0.001, 0.04]	1/minute	[17]
*ρ_tfsh_*	Transfer efficiency of pathogen fromfomite to hands	0.1	[0.01, 0.5]	1/touch	[4], [19]
*ρ_tfhs_*	Transfer efficiency of pathogen fromhands to fomites	0.1	[0.01, 0.5]	1/touch	[4], [19]
*φ_H_*	Fraction of virus shed on hands	0.145	[0.02, 0.48]		

a *α* is the shedding amount per unit time, and *γ* is the recovery rate.

**Table 2:** Fomites Classification Parameters and Derived Parameter Values.

Parameters	Parameter Description	Values					Notes
		Low *λ*, high *ρ_ptr_* (HTSS^a^)	Low *λ*, low *ρ_ptr_* (RTSS^b^)	High *λ*, high *ρ_ptr_* (HTLS^c^)	High *λ*, low *ρ_ptr_* (RTLS^d^)		
*λ*	Proportion of virus that settle on fomites	0.005	0.005	0.145	0.145		Large surface is 29 times larger than small surface
*ρ_ptr_* e	Personal touching rate	0.357	0.018	0.357	0.018		
*ξ*	Proportion of virus shed to handswith reference to the fomite type	0.25	0.25	0.25	0.25		
Derived Parameters							
*ρ_fsr_* f	Ratio of finger area to thesurface area	0.0012	0.0012	4×10^−5^	4×10^−5^	*ρ_fsr = _*0.0012×0.005/*λ*	
*ρ_F_*	Pickup rate	4.28×10^−5^	2.1×10^−6^	1.4×10^−6^	7.2×10^−8^	*ρ_F_* = *ρ_ptr_ ρ_tfsh_ρ_fsr_*	
*δ*	Deposit rate	0.0357	0.0018	0.0357	0.0018	*δ = ρ_ptr_ ρ_tfhs_*	
*P_Deposit_*	Transfer fraction of deposit process	0.0068	0.0004	0.0068	0.0004	*P_Deposit_ = δ ξ*/(*µ_H_ +ρ_inoc_+δ*)	
Parameter groups							
*φ_H_P_Deposit_*		9.8×10^−4^	5×10^−5^	9.8×10^−4^	5×10^−5^		
*λ*(1-*φ_H_*_)_		0.0043	0.0043	0.124	0.124		
*P_Pickup_*		0.8288	0.1736	0.1232	0.0071		
*P_Inoculation_*		0.0608	0.0624	0.0608	0.0624		
Examples		Door handle	Handle of door left open	Table in public place	Rarely used workplace table		

a highly touched small surface.

b rarely touched small surface.

c highly touched large surface.

d rarely touched large surface.

e The sum of *ρ_ptr_* for the four fomite types is 0.75, which is the same value as that in [4].

f Within the venue we assume that there are many realizations of the same type of fomite, and that there are 250 fomites of each type and a population size of 1,000. For a small surface, we assume the ratio of finger area to one of the small fomite surface area is 0.3, therefore the ratio of finger area to the total surface area of the small fomite is 0.0012. The ratio of finger area to the total surface area of the large fomite is 0.0012 divided by 29.

The model captures several processes involved in the fomite mediated transmission.

### Excretion Process

*Iα* is the amount of pathogen shed from compartment *I* to the environment where *α* is the shedding rate. This model ignores particles with post-evaporative diameters less than 10 µm that stay in the air. We assume that all larger particles settle immediately and that the proportion of excreted droplets going directly to hands and that going to surfaces sum to the total large droplet excretion.

### Hand/Fomite Contamination Process

The proportion *φ_H_* of virus that is excreted in the Excretion process goes directly to the hands of an infected individual. The proportion of excreted virus that settles to surfaces immediately, therefore, is 1 - *φ_H_*. Since we defined fomites as touchable surfaces, only a fraction *λ* of pathogens going to surfaces reach one type of fomite. We assume that pathogens settle on surfaces homogeneously, then the fraction *λ* is proportional to the surface area of the fomite. For all pathogens shed into the environment, the proportion (1 – *φ_H_*) *λ* goes to one type of fomite. Hence the shedding rate of pathogens to one type of fomite is *α* (1 – *φ_H_*) *λ*. Because we will include four fomite types in the environment in our later analysis and the four fomite types are assumed to be independent of each other, we assume that the proportion of virus that is excreted to hands and then is associated with a type of fomite is *ξ*. Hence the shedding rate of virus to hands with reference to a type of fomite is *αφ_H_ ξ*.

### Infected Hand Deposition Process

When individuals touch fomites, their contaminated hands deposit pathogens to fomites at a rate *δ*, the product of the individual touching rate (*ρ_ptr_*) and the transfer efficiency of pathogens from hand to fomite per touch (*ρ_tfhs_*).

### Pickup Process

Individuals touch fomites thereby picking up pathogens at the rate *ρ_F_*, the product of the personal touching rate (*ρ_ptr_*), the fraction of the pathogens on a fomite that are touched (*ρ_fsr_*) (assumed to be the ratio of the fingertip area to the total fomite surface area), and the transfer efficiency of pathogens from fomite to hand per touch (*ρ_tfsh_*).

### Self-inoculation Process

Individuals self-inoculate pathogens at the rate *ρ_inoc_* per unit time, transferring pathogens from their hands to their nasal mucosa.

### Infection Process

Each pathogen that reaches the nasal mucosa acts independently of other current or past pathogens on the mucosa to cause infection in susceptible individuals at the risk of *π_F_*.

### Pathogens Die Off Process

Pathogens in the environment are assumed to die off at the rates *µ* *_H_* and *µ* *_F_* for those residing on hands and fomites respectively. Once the viable viruses left the fomite or hands and reached another source then their decay rate changes accordingly.

The two fomite transmission routes involve different sequences of the above processes. The droplet-contaminated-fomite route involves the processes of excretion, fomite contamination, pickup, self-inoculation, pathogen die off, and infection. A redeposition loop of droplet contamination on fomites that are picked up onto hands and then redeposited onto fomites can also be a part of this route. The hand-contaminated-fomite route involves the processes of excretion, hand contamination, infected hand deposition, pickup, self-inoculation, pathogen die off and infection. Again there may be redeposition loop in this route.

The equations are presented below.
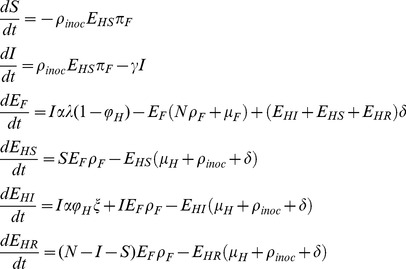
(1)


The equations describe the EITS model with the extension of compartment *E* (living pathogens in the environment) in [15] into four compartments described in the last four equations. The first two equations describe the dynamics of compartments *S*, *I*. The new infections are the result of self-inoculation from contaminated susceptible hands. The infected individual is recovered and become immune at rate *γ* per unit time. The last four equations describe the dynamic of pathogens on fomites and dynamics of pathogens on hands of susceptible, infected and recovered respectively, considering pathogens shed to hands or fomites, pathogens transferred between fomites and hands during pickup and deposition processes, pathogens loss in the self-inoculation process and from virus inactivation.

The above model describes a transmission process defined by one fomite type, *E_F_*, and two fomite contamination routes. In our analysis we examine 4 fomite types by varying two parameters, *λ* and *ρ_ptr_*, as described in the following section. By assuming that the fomite types are independent of each other, a model that includes all 4 fomites can be evaluated by linearly combining the output of the 4 parameterized models.

## Results

We first formulate and interpret the basic reproduction number of our model to illustrate the contributions of the two fomite routes mathematically. Then we categorize fomites into four distinct types based on all dichotomous combinations of high versus low touching rates, and large versus small fomite surface areas. We compare the relative transmission potentials of each fomite route, and finally examine the effect of increasing or decreasing parameter values on the transmission potential for each of the fomite types.

### *R*_0_s of Transmission Routes

The reproduction number for fomite transmission of one fomite type, *R_0_F_*, is characterized by the sum of a droplet-contaminated route *R_0_dF_*, and a hand-contaminated route *R_0_hF_*. (See Appendix S1 for the derivation.).

First, we quantitatively define three processes discussed in the Methods section: *P_Deposit_*, the proportion of viable pathogens reaching hands that are eventually deposited to fomites while still viable.
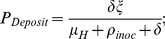

*P_Pickup_*, the proportion of pathogens that arrive on a fomite and are eventually picked up by human hands while still viable,



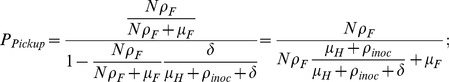
(this formulation incorporates an infinite sequence of pathogen pickup from and redeposition to the fomite.) and *P_Inoculation_*, the proportion of pathogens on hands that are eventually self-inoculated while still viable,



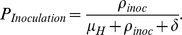



Based on these three processes, the fomite reproduction number of one fomite type can be derived as the following:

Droplet-contaminated route:
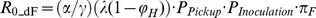
(2.a)


Hand-contaminated route:
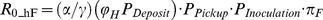
(2.b)


Total:


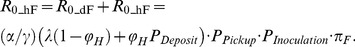
(2.c)

The first factor in equation (2.c) is the total amount of live virus excreted over the course of infection that goes to the fomite: where (*α/γ*) (*λ* (1 *- φ_H_* )) is the amount that deposit directly on fomites, and (*α/γ*)(*φ_H_ P_Deposit_*) is the amount that deposits first on hands of infectious individuals and then on fomites while still viable. The product *P_Pickup_·P_Inoculation_* is the fraction of viruses on fomites that are eventually transferred to the nasal mucosa in a still viable condition.

### Effects of Fomite Characteristics and the Classification of Fomites

**Figure 2:**
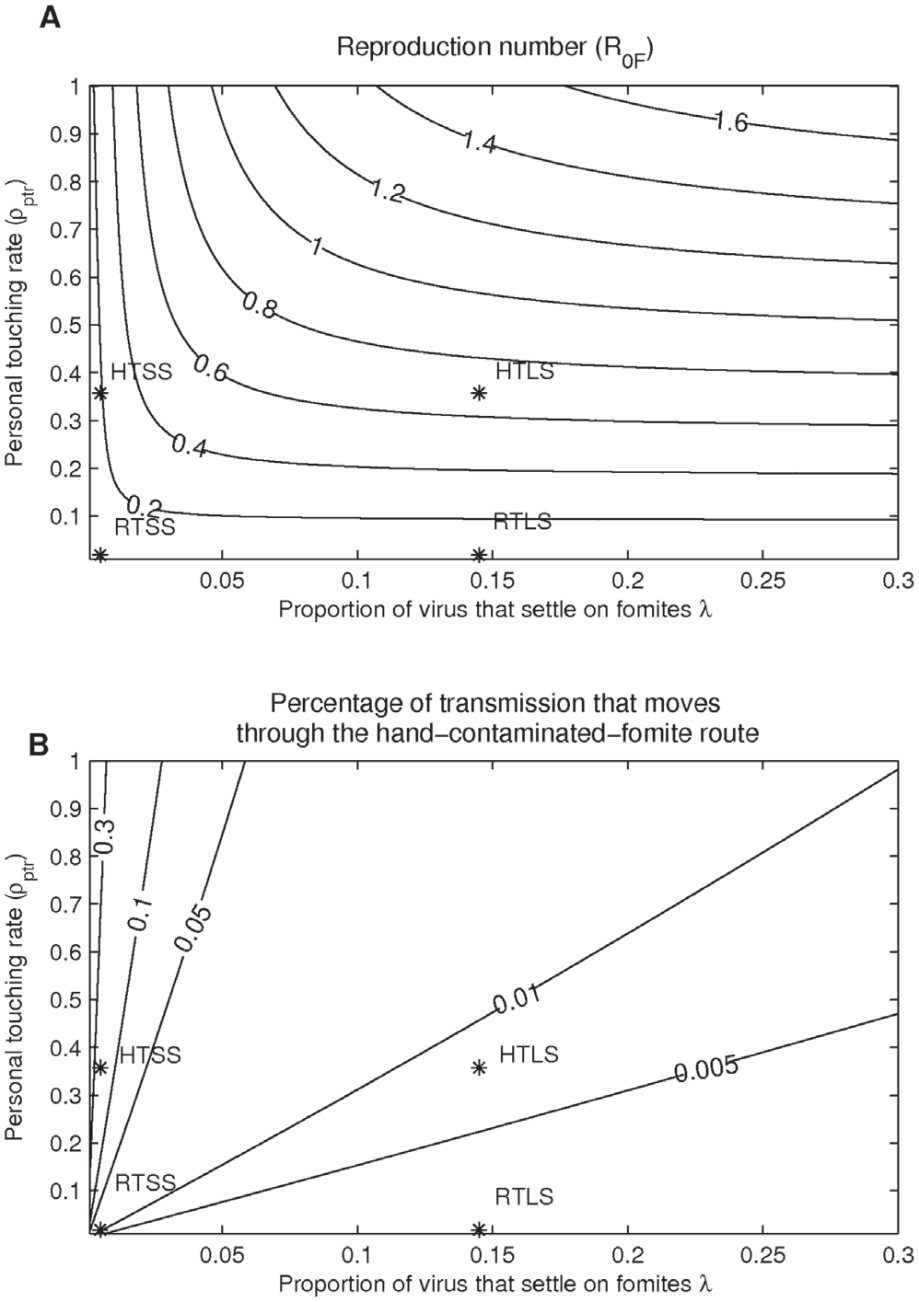
Total transmission potential and the percentage of transmission through hand-contaminated-fomite route. A) total transmission potential (Reproduction number); B) the percentage of transmission through hand-contaminated-fomite route (*R_0_hF_/R_0F_*) as functions of the proportion of virus that settle on fomites (*λ*) and the personal touching rate (*ρ_ptr_*). Stars in the figures are the cases when the parameters take on values in Tables 1 and 2. Changes in *ρ_ptr_* have more impact on the fomite transmission when *ρ_ptr_* is low than when *ρ_ptr_* is high.

The proportion of virus that settles on fomite *λ* and the personal touching rate *ρ_ptr_* are two important parameters involved in fomite transmission. Their effects on fomite transmission are shown in Figure 2∶1) a large surface fomite (higher value of *λ*) has higher *R_0_F_* than a small surface fomite because it can capture more viruses; 2) a highly touched fomite (higher value of *ρ_ptr_*) has higher *R_0_F_* than a rarely touched fomite because more viruses can move through the fomite; 3) a large surface fomite has a lower percentage of transmission through the hand-contaminated-fomite route; 4) a highly touched fomite has higher percentage of transmission through the hand-contaminated-fomite route.

Changes in *ρ_ptr_* have more impact on the fomite transmission when *ρ_ptr_* is low than when *ρ_ptr_* is high. This is because for a given surface size, rarely touched fomites have higher virus concentrations than highly touched fomites. (This can also be observed in equation (5) in Appendix S1, where highly touched surfaces that have a higher *ρ_F_* value have a lower amount of virus on their surface (*E_F_*), and thus have lower virus concentrations.) More viruses are picked up at each touch from rarely touched fomite, and thus the transmission through rarely touched fomite is more sensitive to the changing of the touching rate.

In the following analysis, we dichotomize *λ* and *ρ_ptr_* to classify fomites found in the environment into four types: highly touched large surface (HTLS), rarely touched large surface (RTLS), highly touched small surface (HTSS) and rarely touched small surface (RTSS). A HTLS type of fomite captures a high fraction of shedding virus and is touched frequently, e.g., a table in a public place. An example of the RTLS fomite type is a rarely used table in a workspace, which can capture a lot of droplets and is touched only a few times a day. Examples of the HTSS fomite type are door knobs or elevator buttons that are highly touched but can not catch many droplets. An example of the RTSS fomite type is the handle on a door that is usually left open and therefore only captures a small proportion of shedding virus and is only touched a few times a day. Values of *λ* and *ρ_ptr_* were chosen to illustrate how these fomite pathways contribute to infection; they are not intended to represent any specific environment.

Other fomite characteristics, such as the material type of the fomite and the way a fomite is touched, are not used for the fomite classification, however these characteristics can be captured by one or more parameters in our model. For example, different material types have different values of virus inactivation rates on fomite and transfer efficiencies. Another example is different ways that fomites may be touched: some small surface fomites, such as telephones or handrails on buses, may be held for a relatively long time period with very low touch rate. The fomite that is held longer may have a higher value of *ρ_tfhs_* than those that are held short (for example, elevator button, door knobs.) The effects of these fomite related parameters on fomite transmission will be analyzed in a later section.

Fomites such as eating utensils which will not be repeatedly used in public without being cleaned, or handkerchiefs and tissues which are mainly personal belongings and not used in public, are not considered as public fomites in our model. Use of tissues and handkerchiefs will increase the value of the fraction of virus shed on hands *φ_H_* but may decrease the proportion of virus settling on other fomites *λ*.

Among the four fomite types, the HTLS fomite type accounts for the highest transmission, and the HTSS fomite type accounts for the highest proportion of transmission through the hand routes (*R*_0_hF_/*R*_0_F_) (see stars in Figure 2).

### Comparison of *R*_0_s for the Droplet and Hand Contaminated Fomite Routes

To compare the droplet- and the hand-contaminated-fomite routes of a fomite type, we examine the ratio of the *R_0_* expressions. Parameter values used in the comparison primarily come from the literature and our prior publications [4], [15]. There is no specific study of the fraction of virus excreted to hands, and its value can depend on social behavior, culture, and personal habits etc. In some model analyses of transmission involving fomites, viruses are assumed to be shed onto the fomites [6], [16], but contamination via infected individuals’ hands is not modeled. We assume a value of *φ_H_* to be 0.145, reflecting the situation where the majority of shedding due to coughing or sneezing is not caught by the hand. Since value of *ξ* does not affect our conclusion, we assume *ξ* to be 0.25 for each fomite type. The shedding amount, *α/γ*, was set to 1×10^7^ pathogens/infected so that *R*_0_ values range from 0.1 to just greater than 1. Finally, the population size and the total size of fomites that is used to calculate the ratio of finger area to the surface area (*ρ_fsr_*) are related to the space of the venue; a larger venue has a higher population and larger total fomite size. We use 1,000 individuals as our population size, and fomite sizes are specified correspondingly in Table 2.

From equations 2a and 2b, we observe that three processes (pickup, self-inoculation, and infection) as well as average shedding amount appear linearly in each *R*_0_ expression and therefore cancel out when examining the ratio:
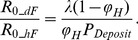
(3)


The transmission percentage that moves through the hand-contaminated-fomite route is a complementary way to look at the comparison of hand and droplet contaminated fomite routes:
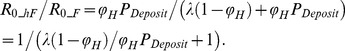
(4)


In the above equations, the relative transmission potentials of the two fomite routes on a fomite type are only affected by the fraction of droplets that settle to the fomite (*λ*), the fraction of hand contamination that reaches fomites while still viable (*P_Deposit_*) and the proportion of virus shed to hands directly (*φ_H_*). The parameter *λ* relates to the size of the fomite and reflects its droplet contamination level. The touch frequency of a fomite (*ρ_ptr_*) positively affects *P_Deposit_*. As *λ* increases, more viruses move through the droplet-contaminated-fomite route; as *ρ_ptr_* increases, more viruses move through the hand-contaminated-fomite route. In either case, *R*_0_F_ increases.

**Figure 3:**
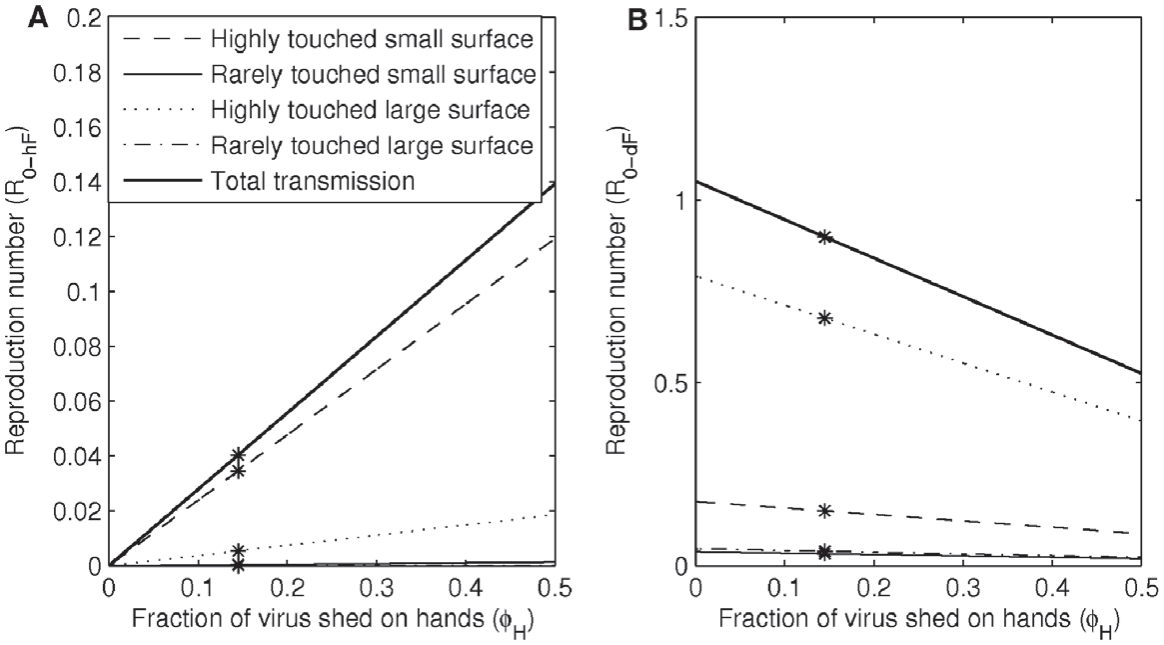
Transmission potentials (Reproduction number). A) Reproduction number for the hand-contaminated route (*R_0_hF_*) as functions of the fraction of virus shed on hands; B) reproduction number for the droplet-contaminated fomites route (*R_0_dF_*) as functions of the fraction of virus shed on hands. Note that scale for graph A) is one order of magnitude smaller than graph B). Values are shown for all fomites combined and individual fomite types. The upper limit of x-axis (0.5) is based on the assumption that the fraction shed to hands is less than that shed into air. The parameters for each of the four fomite classes graphed are presented in Tables 1 and 2. Stars in A and B are R0s of the two fomite routes when *φ_H_* = 0.145.

A parameter that strongly affects both hand and droplet contaminated fomite routes is the fraction of virus shed on hands, *φ_H_* (Figure 3). For all fomite types, as *φ_H_* increases, *R_0_hF_* increases and *R_0_dF_* decreases; however except for the HTSS fomite type, in general more than 90% of the transmission is accounted for by the droplet-contaminated-fomite route. For the HTSS fomite type, when *φ_H_* approaches 0.42 (42% of virus shedding goes to the hands), both routes account for similar amounts of transmission.

### Parameter Effects on the Combined Fomite Route Transmission

**Figure 4:**
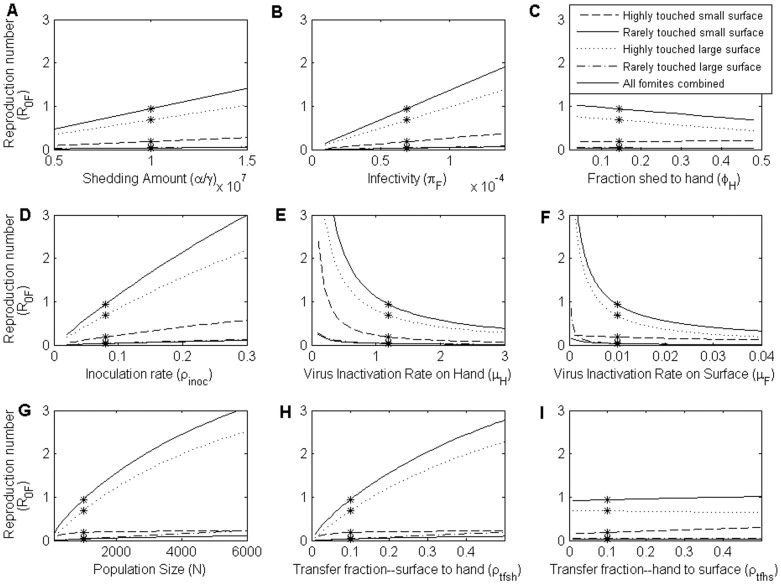
The total fomite transmission potentials. Total fomite transmission potential (Reproduction number) as a function of A) shedding amount *α/γ*, B) dose response of virus on mucosa *π_F_*, C) fraction of virus shed on hands *φ_H_*, D) inoculation rate *ρ_inoc_*, E) virus inactivation rate on hands *µ* *_H_*, F) virus inactivation rate on fomites *µ* *_F_*, G) population size *N*, H) fraction of pathogen transferred from surface to hands *ρ_tfsh_*, and I) fraction of pathogen transferred from hands to surface *ρ_tfhs_*, stratefied by fomite types. Stars on the lines are values of *R_0_F_* when the parameters take on values in Tables 1 and 2. Across all variation considered, the highly touched large surface fomite type has the highest transmission potential. The slope of each line, which can be interpreted as strength of each hypothetical control, is largest for the highly touched large surface fomite type.

Sensitivity analysis was conducted on 9 parameters listed in Table 1 and Figure 4. Values used for *φ_H_ P_Deposit_*, *λ* (1 - *φ_H_*), *P_Pickup_* and *P_Inoculation_* for the four fomite types are listed in Table 2 for comparison.

By observing the slopes of the relationship between *R*_0_F_ of the four fomite types and various parameters, we find that in general the HTLS fomite type strongly affects the strength of the fomite transmission (Figure 4). To examine the impact of interventions parameters were varied. The parameters can be increased or decreased to reflect intervention measures against fomite transmission. For example, increasing *µ* *_H_* or *µ* *_F_* represents hand washing or fomite decontamination. Hence interventions such as washing hands, cleaning fomites, inoculating less, decreasing shedding amount and keeping population size small, would be most effective against the transmission through the HTLS fomite type. Among these parameters, the self-inoculation rate *ρ_inoc_*, the population size *N* and the transfer fraction from surface to hands *ρ_tf_sh_* have high impact, and the transfer fraction from hand to surface *ρ_tfhs_* has the lowest impact (Figure 4I), on the fomite transmission.

Parameters that are only related to the infected compartment or infectious sites of susceptibles, such as shedding amount (*α/γ*) and infectivity (*π_F_*), are linearly related to the fomite transmission; in addition, fraction shed to hands (*φ_H_*) and self-inoculation rate (*ρ_inoc_*) are approximately linearly related to the fomite transmission (Figures 4A–D). Behavioral interventions that can reduce shedding amount or self-inoculation frequency constantly reduce fomite transmission. Examples of behavioral interventions that can reduce the shedding amount (or remove virus from the system) are: having infected individuals cough or sneeze into the clothing on their upper arm assuming this clothing is not touched by others; having infected individuals shed on tissues that are then discarded.

Increasing *φ_H_* can have a positive or negative effect on *R*_0_F._ We can see this by rewriting *R*_0_F_ as follows:
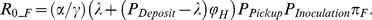
(5)


The term *P_Deposit –_ λ* can be positive or negative depending on whether *λ* <*P_Deposit_* or *λ*>*P_Deposit_*. The slope of the relationship between *R*_0_F_ and *φ_H_*, therefore, can be positive or negative. *λ* is less than *P_Deposit_* for the HTSS fomite type and larger than *P_Deposit_* for the other three fomite types. Covering coughs and sneezes by hands (increasing *φ*) therefore increases the fomite transmission for the HTSS fomite type and decreases the fomite transmission for the other three fomite types. Shedding on tissues could increase the virus fraction going to hands (*φ_H_*) while decreasing the shedding amount (*α/γ)*. For the HTSS fomite type, the effect of decreasing *α/γ* can largely override the effect of increasing *φ_H_*. For the other three fomite types, transmission potentials are further reduced in addition of those reduced from decreasing *α/γ*.

Parameters related to the environment, such as touching rate (*ρ_ptr_*), virus inactivation rate on hands (*µ* *_H_*) and fomites (*µ* *_F_*), population size (*N*) and transfer fraction from surface to hands (*ρ_tfsh_*), have greater impact on fomite transmission in low values of the range considered (Figures 4E–H). Environmental interventions like washing hands or fomite decontamination are therefore more effective when the related parameter *µ* *_H_* or *µ* *_F_* is in the low parameter spectrum. Because the influenza virus is characterized by a *µ* *_H_* value that ranges from around 0.92 to 1.47 per minute [17], washing hands may not be as effective at decreasing influenza transmission as the same intervention against transmissions of viruses or bacteria that live longer on human hands, for example S. aureus. Similarly, because different fomite materials have different *µ* *_F_* values (*µ* *_F_* for a nonporous fomite is 0.002/minute and *µ* *_F_* for a porous fomite is 0.016/minute), a fomite decontamination intervention of a nonporous material like a door knob will more efficiently reduce the influenza transmission than a fomite decontamination of a porous material like a bed sheet.

**Figure 5:**
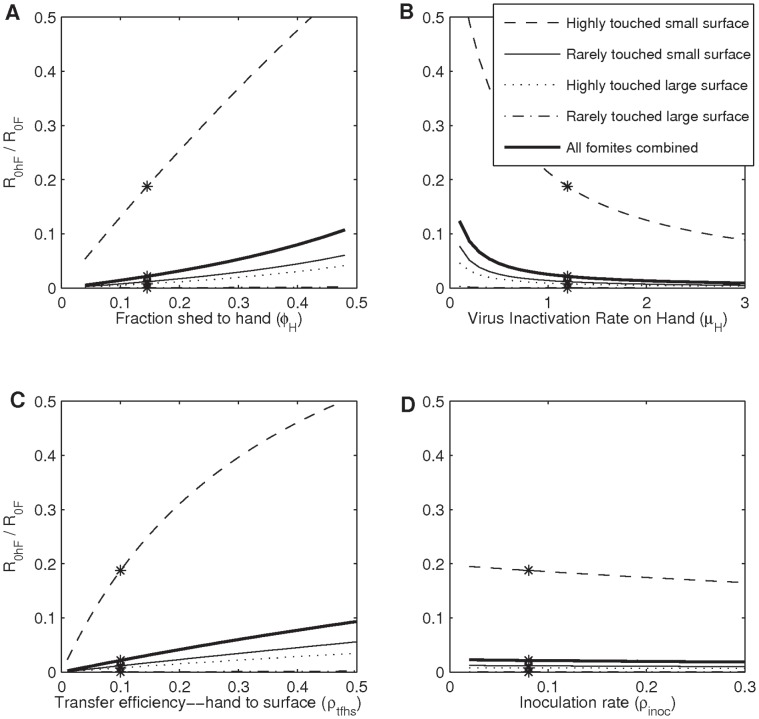
The proportion of all transmission mediated through the hand-contaminated-fomite route. The proportion of all transmission mediated through the hand-contaminated-fomite route as a function of A) fraction of virus shed on hands *φ_H_*, B) virus inactivation rate on hands *µ* *_H_*, C) fraction of pathogen transferred from hands to surface *ρ_tfhs_* and D) inoculation rate *ρ_inoc_*, stratified by fomite type. Stars on the lines are values of *R_0_F_* when the parameters take on values in Tables 1 and 2. The percentage of hand-contaminated-fomite route does not exceed 50% except when the fraction shed to hand is larger than 42% or when the virus inactivation rate on hands is less than 0.2/min or when the virus transfer fraction from hand to surface is larger than 48%.

Figure 5 shows parameter effects on the relative contributions of hand-contaminated-fomite route. For the HTLS, RTLS and RTSS fomites types, the droplet contamination level (*λ* (1 - *φ_H_*)) is much higher than the contamination level from touching (*φ_H_P_Deposit_*). Thus, the transmission through these three fomite types predominantly occurs through the droplet-contaminated-fomite route (*R*_0_hF_/*R*_0_F_<<0.5). On the other hand, the ratio of the HTSS fomite type can be larger than 0.5 when the total fraction shed to hand is very high (>42%), or virus inactivation rate on hands is very low (<0.2/min), or the transfer fraction from hand to surface is very high (>48%). The relative contribution of the hand-contaminated-fomite routes of the venue is examined by the ratio of the sum of *R*_0_hF_ of the four fomite types and the sum of *R*_0_F_ of the four fomite types. Therefore the conditions where hand-contaminated-fomite route contributes more to the total fomite mediated transmission is when a venue mainly consists of HTSS fomites, and infected individuals shed a lot on hands.

The ratio *R*_0_hF_/*R*_0_F_ of the HTSS fomite type is more sensitive to these parameters than the other three fomite types because of the same reason mentioned above.

## Discussion

The Environmental Infection Transmission System (EITS) approach extended from [15] allows for the integration of considerable experimental and theoretical knowledge regarding pathogen survival, transport, and transfer between fomites and hands. This model, like all models, is an oversimplification that helps us to develop new concepts. It creates an initial framework for assessing modes of transmission in the real world and provides a framework for further advances. To advance both theory and practical assessment of the roles of fomite and aerosol transmission in real world settings the unrealistic simplifying assumptions in our model will need to be realistically relaxed. That process should suggest ways that new data can be found to better test and elaborate model assumptions.

The model presented here accounts for one fomite at a time and reveals that for influenza, droplet contamination of fomites is more important than hand contamination of fomites. Realistic complexities such as transport of pathogens from one type of fomite to another may change the relationships we present. They are unlikely, however, to change our conclusion about the source of fomite contamination for influenza and they do not negate the theoretical foundation of this model formulation as a base on which to build more realistic models.

The model also creates a basis for comparing how we might expect transmission dynamics via fomites to change for infectious agents that have parameter values differing from those we have used to characterize influenza. Pathogens with greatly increased hand and environmental survival than is the case for influenza, for example methicillin resistant Staphylococcus aureaus (MRSA), can be expected to have quite different dynamics with quite different relative importance of hand- and droplet-contaminated-fomite routes.

This model improves our understanding of the transmission process not only across fomite routes, but also across fomite types. Here pickup rates and fomite sizes were used to classify fomites. Large surface and highly touched fomites are the most important fomite type because most transmission operates through them; in addition, these fomites are sensitive to most of the interventions examined. Transmissions via other fomite types, however, should not be neglected. Highly touched small surface fomite is relatively ‘clean’, however this cleanliness is due to viruses being picked up by population rapidly; rarely touched fomites have relatively higher virus concentration than highly touched fomites, and each touch of such fomites transmits more virus to susceptibles’ hands.

The parameter sensitivity analysis presented reveals how parameter value estimates affect inferences regarding the effectiveness of intervention measures such as hand washing and fomite decontamination. Specific values of virus inactivation on hands and fomites (x-axis values of stars in Figure 4) are based on only one study in 1982 [17] which reported that influenza viruses can remain viable on hand for up to a minute and surfaces for up to hours, however we extended the values 2 to 10 times larger or smaller to set the parameter rage for the sensitivity study. The effectiveness of hand washing or fomite decontamination is quite different when these parameter values are much smaller or higher than the specific values. The effects of environmental conditions on the inactivation rates of influenza virus on fomites have been studied experimentally, including effects of humidity, temperature [20]; other factors such as UV radiation may also play a role in the changing of inactivation rates of influenza virus on fomites. Effects of varying inactivation rates of virus due to humidity or temperature can be checked using the figures of the sensitivity analysis. Future studies to replicate the results or to have more precise parameter values are suggested for better inference related to the effectiveness of hand washing and fomite decontamination.

Intervening either at shedding (such as coughing into one’s clothes or on a tissue) or at self-inoculation (such as touching one’s mucous membrane less), which are the two ends of the transmission chain, would consistently decrease fomite transmission. Even though we do not have the exact value of the relevant parameters, we can conclude that the greater the population compliance with the above behavioral interventions, the more the transmission decreases and the greater the effectiveness of interventions. On the other hand, interventions related to the environment are subject to diminishing returns whereby, for example, effectiveness of hand washing or fomite decontamination depend on the fomite type and would receive less added benefit given better intervention compliance.

Our extended EITS model assumes that the population size is infinite and the fomite is touched continuously without any touching sequence. In reality, factors such as small population size and event sequences can have large impacts on influenza transmission. For example, in a scenario of a small population size and when an individual is infected only once, touching sequence will change the relative transmission importance of fomite types. These elements cannot be included in our ODE model, but should be addressed by future work using a stochastic or individual based simulation model. Other unrealistic assumptions like instantaneous and homogeneous distribution of viruses on fomites and homogeneous touching of fomites by the whole population, should be relaxed in future work. Future studies should also include the aerosol route together with fomite routes and interactions of fomite types.

The four fomite types classified here are arbitrary. However conclusions from these fomite types hold across a broad range of parameter values. It would be very useful to collect data on fomite type distributions in real venues by careful measurements and observations. This would allow for measurement of fomite characteristics to help assess model performance for capturing transmission in venues.

## Supporting Information

Appendix S1 **The derivation of the reproduction number for the fomite transmission route.**
(DOC)
