# Supplementary material for: Model Analysis of Fomite Mediated Influenza Transmission
Source: PLoS One. 2012 Dec 27;7(12):e51984. doi: 10.1371/journal.pone.0051984 (PMC3531458; doi:10.1371/journal.pone.0051984)
Supplement: Appendix S1 — The derivation of the reproduction number for the fomite transmission route. (DOC) [file pone.0051984.s001.doc]

Appendix S1

*R*_0_ of one fomite type is derived as follows:

Assume that environmental equilibration is much more rapid than population infection dynamics, hence we let the right hand side of the last four equations of equation set (1) in the main text to be zero

$$\begin{matrix} I\alpha\lambda(1-\phi_{H})-E_{F}\left(N\rho_{F}+\mu_{F}\right)+\left(E_{HI}+E_{HS}+E_{HR}\right)\delta=0 \\ SE_{F}\rho_{F}-E_{HS}\left(\mu_{H}+\rho_{inoc}+\delta\right)=0 \\ I\alpha\phi_{H}\xi+IE_{F}\rho_{F}-E_{HI}\left(\mu_{H}+\rho_{inoc}+\delta\right)=0 \\ RE_{F}\rho_{F}-E_{HR}\left(\mu_{H}+\rho_{inoc}+\delta\right)=0 \end{matrix}$$

Then we have,

$E_{F}=\frac{I\alpha\lambda(1-\phi_{H})}{N\rho_{F}+\mu_{F}}+\delta\frac{E_{HI}+E_{HS}+E_{HR}}{N\rho_{F}+\mu_{F}}\text{ }$ (S1)

$E_{HS}=\frac{S\rho_{F}}{\mu_{H}+\rho_{inoc}+\delta}E_{F}\text{ }$ (S2)

$E_{HI}=\frac{I\alpha\phi_{H}\xi}{\mu_{H}+\rho_{inoc}+\delta}+\frac{I\rho_{F}}{\mu_{H}+\rho_{inoc}+\delta}E_{F}$ (S3)

$E_{HR}=\frac{R\rho_{F}}{\mu_{H}+\rho_{inoc}+\delta}E_{F}$ (S4)

Substitute (S2), (S3) and (S4) into (S1), we have

$E_{F}=\frac{\frac{1}{N\rho_{F}+\mu_{F}}I\alpha\lambda(1-\phi_{H})}{1-\frac{N\rho_{F}}{N\rho_{F}+\mu_{F}}\frac{\delta}{\mu_{H}+\rho_{inoc}+\delta}}+\frac{\frac{1}{N\rho_{F}+\mu_{F}}\frac{\delta}{\mu_{H}+\rho_{inoc}+\delta}I\alpha\phi_{H}\xi}{1-\frac{N\rho_{F}}{N\rho_{F}+\mu_{F}}\frac{\delta}{\mu_{H}+\rho_{inoc}+\delta}}$ (S5)

Then substitute the above equation of *E_F_* into (S2),

$\begin{matrix} E_{HS}=\frac{S\rho_{F}}{\mu_{H}+\rho_{inoc}+\delta}\frac{\frac{1}{N\rho_{F}+\mu_{F}}I\alpha\lambda(1-\phi_{H})}{1-\frac{N\rho_{F}}{N\rho_{F}+\mu_{F}}\frac{\delta}{\mu_{H}+\rho_{inoc}+\delta}} \\ +\frac{S\rho_{F}}{\mu_{H}+\rho_{inoc}+\delta}\frac{\frac{1}{N\rho_{F}+\mu_{F}}\frac{\delta\xi}{\mu_{H}+\rho_{inoc}+\delta}I\alpha\phi_{H}}{1-\frac{N\rho_{F}}{N\rho_{F}+\mu_{F}}\frac{\delta}{\mu_{H}+\rho_{inoc}+\delta}}\text{ } \end{matrix}$ (S6)

Substitute (S6) into

$$\frac{dI}{dt}=\rho_{inoc}E_{HS}\pi_{F}-\gamma I$$

An epidemic occurs if *dI/dt* >0, that is

$$\begin{matrix} \frac{S\rho_{F}\rho_{inoc}\pi_{F}}{\mu_{H}+\rho_{inoc}+\delta}\frac{\frac{1}{N\rho_{F}+\mu_{F}}I\alpha\lambda(1-\phi_{H})}{1-\frac{N\rho_{F}}{N\rho_{F}+\mu_{F}}\frac{\delta}{\mu_{H}+\rho_{inoc}+\delta}} \\ +\frac{S\rho_{F}\rho_{inoc}\pi_{F}}{\mu_{H}+\rho_{inoc}+\delta}\frac{\frac{1}{N\rho_{F}+\mu_{F}}\frac{\delta\xi}{\mu_{H}+\rho_{inoc}+\delta}I\alpha\phi_{H}}{1-\frac{N\rho_{F}}{N\rho_{F}+\mu_{F}}\frac{\delta}{\mu_{H}+\rho_{inoc}+\delta}}>\gamma I\text{ } \end{matrix}$$

*I* is canceled on both side. Let *S*=*N*,

$$\begin{matrix} \frac{\alpha\lambda(1-\phi_{H})\pi_{F}}{\gamma\text{ }}\cdot\frac{\frac{N\rho_{F}}{N\rho_{F}+\mu_{F}}}{1-\frac{N\rho_{F}}{N\rho_{F}+\mu_{F}}\frac{\delta}{\mu_{H}+\rho_{inoc}+\delta}}\cdot\frac{\rho_{inoc}}{\mu_{H}+\rho_{inoc}+\delta} \\ +\frac{\alpha\phi_{H}\pi_{F}}{\gamma\text{ }}\frac{\delta\xi}{\mu_{H}+\rho_{inoc}+\delta}\frac{\frac{N\rho_{F}}{N\rho_{F}+\mu_{F}}}{1-\frac{N\rho_{F}}{N\rho_{F}+\mu_{F}}\frac{\delta}{\mu_{H}+\rho_{inoc}+\delta}}\frac{\rho_{inoc}}{\mu_{H}+\rho_{inoc}+\delta}>1 \end{matrix}$$

Then

$$\begin{matrix} R_{0}=\frac{\alpha\lambda(1-\phi_{H})\pi_{F}}{\gamma\text{ }}\cdot\frac{\frac{N\rho_{F}}{N\rho_{F}+\mu_{F}}}{1-\frac{N\rho_{F}}{N\rho_{F}+\mu_{F}}\frac{\delta}{\mu_{H}+\rho_{inoc}+\delta}}\cdot\frac{\rho_{inoc}}{\mu_{H}+\rho_{inoc}+\delta} \\ +\frac{\alpha\phi_{H}\pi_{F}}{\gamma\text{ }}\frac{\delta\xi}{\mu_{H}+\rho_{inoc}+\delta}\frac{\frac{N\rho_{F}}{N\rho_{F}+\mu_{F}}}{1-\frac{N\rho_{F}}{N\rho_{F}+\mu_{F}}\frac{\delta}{\mu_{H}+\rho_{inoc}+\delta}}\frac{\rho_{inoc}}{\mu_{H}+\rho_{inoc}+\delta} \end{matrix}$$

or

$$\begin{matrix} R_{0\_dF}=\frac{\alpha\lambda(1-\phi_{H})\pi_{F}}{\gamma\text{ }}\cdot\frac{\frac{N\rho_{F}}{N\rho_{F}+\mu_{F}}}{1-\frac{N\rho_{F}}{N\rho_{F}+\mu_{F}}\frac{\delta}{\mu_{H}+\rho_{inoc}+\delta}}\cdot\frac{\rho_{inoc}}{\mu_{H}+\rho_{inoc}+\delta} \\ R_{0\_hF}=\frac{\alpha\phi_{H}\pi_{F}}{\gamma\text{ }}\frac{\delta\xi}{\mu_{H}+\rho_{inoc}+\delta}\frac{\frac{N\rho_{F}}{N\rho_{F}+\mu_{F}}}{1-\frac{N\rho_{F}}{N\rho_{F}+\mu_{F}}\frac{\delta}{\mu_{H}+\rho_{inoc}+\delta}}\frac{\rho_{inoc}}{\mu_{H}+\rho_{inoc}+\delta} \end{matrix}$$

Denote

$$P_{Deposit}=\frac{\delta\xi}{\mu_{H}+\rho_{inoc}+\delta}$$

$$P_{Inoculation}=\frac{\rho_{inoc}}{\mu_{H}+\rho_{inoc}+\delta}$$

$$P_{Pickup}=\frac{\frac{N\rho_{F}}{N\rho_{F}+\mu_{F}}}{1-\frac{N\rho_{F}}{N\rho_{F}+\mu_{F}}\frac{\delta}{\mu_{H}+\rho_{inoc}+\delta}}$$

*P_Pickup_* is the result of infinite series of all touching (pickup and deposit) processes. For this geometric series, the first value is $\frac{N\rho_{F}}{N\rho_{F}+\mu_{F}}$, and common ratio is $\frac{N\rho_{F}}{N\rho_{F}+\mu_{F}}\frac{\delta}{\mu_{H}+\rho_{inoc}+\delta}$. *P_Pickup_* can be simplified as

$$\begin{matrix} P_{Pickup}=\frac{\frac{N\rho_{F}}{N\rho_{F}+\mu_{F}}}{1-\frac{N\rho_{F}}{N\rho_{F}+\mu_{F}}\frac{\delta}{\mu_{H}+\rho_{inoc}+\delta}}=\frac{N\rho_{F}}{N\rho_{F}+\mu_{F}-N\rho_{F}\frac{\delta}{\mu_{H}+\rho_{inoc}+\delta}} \\ =\frac{N\rho_{F}}{N\rho_{F}\frac{\mu_{H}+\rho_{inoc}}{\mu_{H}+\rho_{inoc}+\delta}+\mu_{F}} \end{matrix}$$

then

$$R_{\text{0\_dF}}=\left(\alpha/\gamma\right)\left(\lambda(1-\phi_{H})\right)\cdot P_{Pickup}\cdot P_{Inoculation}\cdot\pi_{F}$$

$$R_{\text{0\_hF}}=\left(\alpha/\gamma\right)\left(\phi_{H}P_{Deposit}\right)\cdot P_{Pickup}\cdot P_{Inoculation}\cdot\pi_{F}$$

$$R_{0\_F}=R_{\text{0\_dF}}+R_{\text{0\_hF}}=\left(\alpha/\gamma\right)\left(\lambda(1-\phi_{H})+\varphi_{H}P_{Deposit}\right)\cdot P_{Pickup}\cdot P_{Inoculation}\cdot\pi_{F}$$
